# Crosstalk between P2Y receptors and cyclooxygenase activity in inflammation and tissue repair

**DOI:** 10.1007/s11302-023-09938-x

**Published:** 2023-04-13

**Authors:** Adrián Povo-Retana, Sergio Sánchez-García, Carlota Alvarez-Lucena, Rodrigo Landauro-Vera, Patricia Prieto, Carmen Delgado, Paloma Martín-Sanz, Lisardo Boscá

**Affiliations:** 1 Instituto de Investigaciones Biomédicas Alberto Sols (Centro Mixto CSIC-UAM), Arturo Duperier 4, 28029 Madrid, Spain; 2 Departamento de Farmacología, Farmacognosia y Botánica. Facultad de Farmacia, Universidad Complutense de Madrid, Plaza Ramón y Cajal, 28040 Madrid, Spain; 3 Centro de Investigación Biomédica en Red en Enfermedades Cardiovasculares (CIBERCV), Melchor Fernández Almagro 6, 28029 Madrid, Spain; 4 Centro de Investigación Biomédica en Red de Enfermedades Hepáticas y Digestivas (CIBEREHD), Melchor Fernández Almagro 6, 28029 Madrid, Spain

**Keywords:** Purinergic receptor, Prostaglandin, Macrophage, Protein kinase C, Protein kinase D

## Abstract

The role of extracellular nucleotides as modulators of inflammation and cell stress is well established. One of the main actions of these molecules is mediated by the activation of purinergic receptors (P2) of the plasma membrane. P2 receptors can be classified according to two different structural families: P2X ionotropic ion channel receptors, and P2Y metabotropic G protein-coupled receptors. During inflammation, damaged cells release nucleotides and purinergic signaling occurs along the temporal pattern of the synthesis of pro-inflammatory and pro-resolving mediators by myeloid and lymphoid cells. In macrophages under pro-inflammatory conditions, the expression and activity of cyclooxygenase 2 significantly increases and enhances the circulating levels of prostaglandin E_2_ (PGE_2_), which exerts its effects both through specific plasma membrane receptors (EP1-EP4) and by activation of intracellular targets. Here we review the mechanisms involved in the crosstalk between PGE_2_ and P2Y receptors on macrophages, which is dependent on several isoforms of protein kinase C and protein kinase D1. Due to this crosstalk, a P2Y-dependent increase in calcium is blunted by PGE_2_ whereas, under these conditions, macrophages exhibit reduced migratory capacity along with enhanced phagocytosis, which contributes to the modulation of the inflammatory response and tissue repair.

## Inflammation specificities and factors involved

The regulation of the inflammatory response remains a central aspect in the understanding of many pathological processes [1–6]. The three phases that characterize inflammation, i.e., initiation, extension, and repair/resolution, are controlled by a large number of factors with specific temporal and intensity patterns [7, 8]. These profiles vary between tissues and species, defining the course of the pathological process and the impact on the organisms [9]. However, despite the selectivity of many inflammatory reactions, there is an overlap in the molecular pathways involved. This diversity in the interactions between them defines specific fates in their control and the possible therapeutic interventions [10–12]. An example of this is the involvement of P2X_7_ receptor signaling in the activation of the NLRP3 inflammasome, which requires the involvement of an additional priming signal from the TLR2/4 pathway [13–17].

**Fig. 1:**
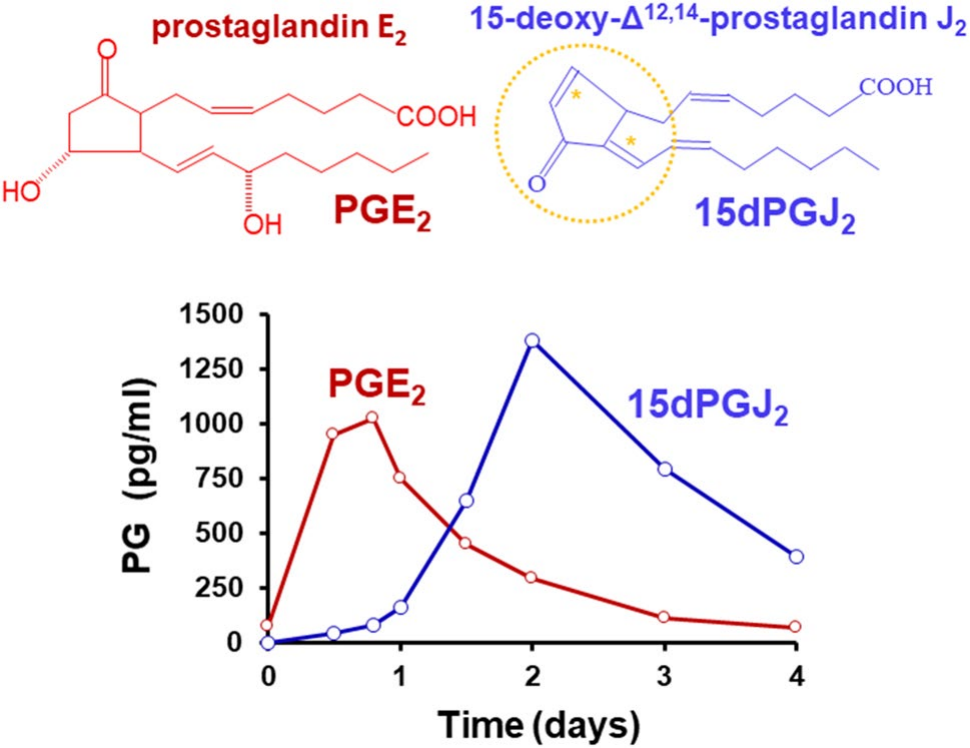
Time course of the serum levels of PGE_2_ and 15-deoxy-Δ.^12,14^-PGJ_2_ (15dPGJ_2_) in mice injected intraperitoneally with LPS. 12-month-old male mice (*n* = 7) received 1 mg/kg body weight of *E. coli* LPS (serotype 0055:B5) and the serum levels of early pro-inflammatory (PGE_2_) and anti-inflammatory (15dPGJ_2_) prostaglandins were determined using specific ELISA kits. The graph represents the mean values and shows a minimal overlapping of both prostaglandins (unpublished results from the authors). The structures of PGE_2_ and 15dPGJ_2_ are shown. 15dPGJ_2_ has a cyclopentenone structure which is responsible for its reactivity to perform Michael addition reactions on thiol groups from amino acids (a carbonyl group surrounded by α, β instaurations on the cyclopentenone ring; yellow stars and circle)

**Fig. 2:**
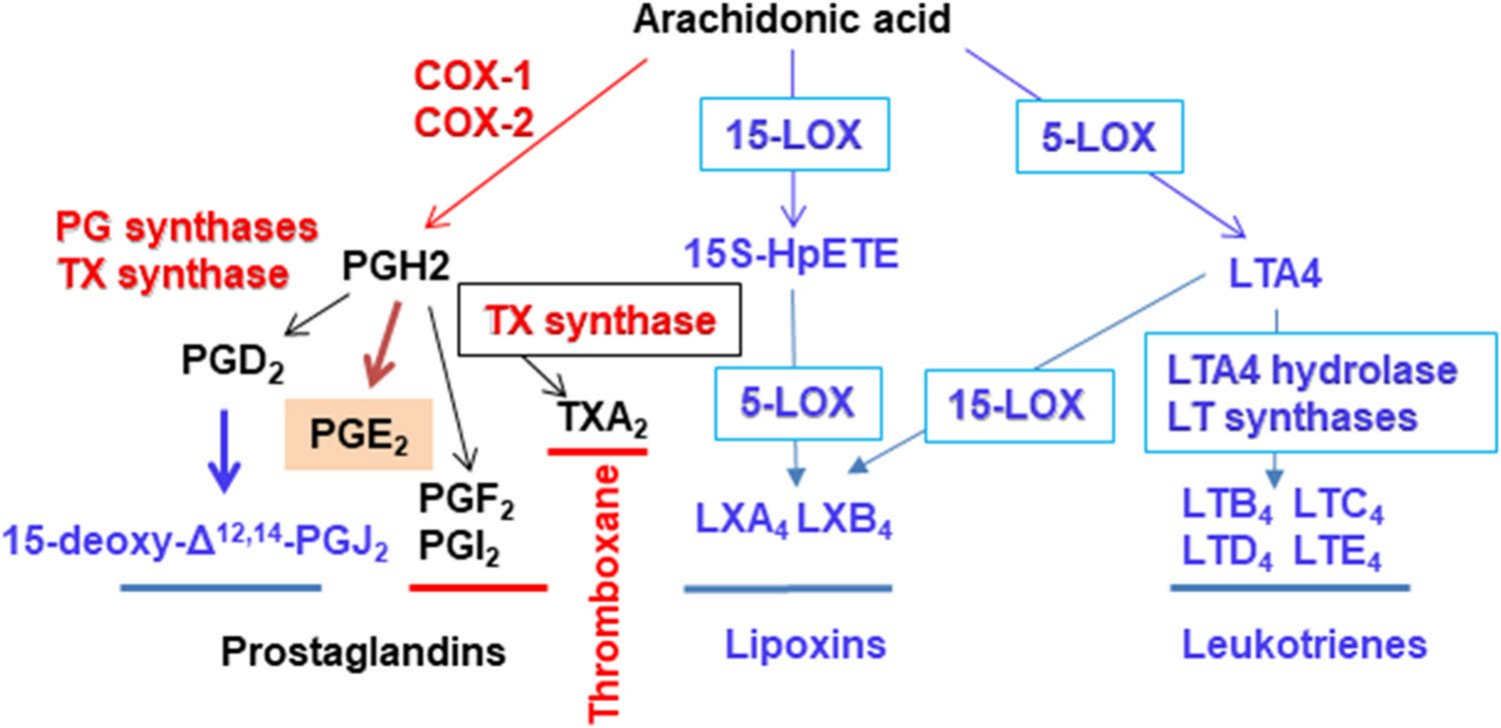
Schematic representation of the synthesis of PGs and pro-resolving lipids from arachidonic acid. Cyclooxygenases (COX-1 and COX-2) and lipoxygenases (5-LOX and 15-LOX) are the initial enzymes that direct the biotransformation of the arachidonic acid after activation of plasma membrane phospholipases. *Red names/lines*, the main pro-inflammatory molecules; *blue names/lines*, the main anti-inflammatory/pro-resolving molecules; in *black* the molecules that play a dual role in inflammation. TX, thromboxane; LT, leukotriene; LX, lipoxin; PGF_2_, prostaglandin F_2α_ (also known as dinoprost); PGI_2_, prostacyclin; 15*S*-HpETE, 15-hydroperoxyicosa-5,8,11,13-tetraenoic acid

It is worth mentioning that the production of different bioactive lipids, such as prostanoids, is a common determinant in the progression of inflammatory processes (Fig. 1) [18–20]. The most abundant prostanoids from pro-inflammatory macrophages are synthesized after the expression of cyclooxygenase 2 (COX-2), which catalyzes the first step in the biosynthesis of prostanoids from arachidonic acid [21–28]. The end products of the COX-2 pathway are the result of additional modifications *via* the action of cell-specific prostaglandin synthases (Fig. 2) [29]. COX-2 is encoded by the *PTGS2* gene in humans (*Ptgs2* in rodents) and it is expressed in the early stages of inflammation. The transcription of the *PTGS2* gene is extensively induced in many inflammatory cells and tissues, except in hepatocytes, where only after preliminary pathological changes (i.e., liver regeneration after partial hepatectomy) is the ability to express COX-2 recovered [26, 30, 31]. In the liver, this regulatory bias is only associated with hepatocytes, since Kupffer cells retain this pro-inflammatory activation [28, 32–37]. This interesting mechanism reflects the fact that, under physiological conditions, the portal blood contains pathogen-associated molecular patterns (PAMPs) and damage-associated molecular patterns (DAMPs), which do not activate COX-2 expression through cell surface receptors that recognize PAMP or DAMP [38, 39].

## Dual role of prostaglandins in the regulation of inflammation

The prostanoids synthesized by the COX-2 pathway can act in opposite ways: they can exert pro-inflammatory actions, but they can also promote and activate anti-inflammatory mechanisms [40–43]. An example of this dual role is PGE_2_, which is one of the major products of the COX-2 pathway [44–50]. Other prostaglandins, such as prostaglandin 15-deoxy-Δ^12,14^-prostaglandin J_2_ (15dPGJ_2_) are potent anti-inflammatory molecules because their chemical structure contains a cyclopentenone motif (due to the presence of α,β-unsaturated carbonyl groups). This chemical structure allows for non-enzymatic reactions with cysteine residues in proteins, via Michael addition modifications (Figs. 1 and 2) [40, 51–53]. These Michael adducts have an impact on the enzyme activity and function of different proteins involved in the control of the inflammatory processes, such as the transcription factor NF-κB, which exerts an important activation of the pro-inflammatory response, and is inhibited by Michael addition of 15dPGJ_2_ [54]. In contrast, transcription factors that repress the progression of inflammation, such as the peroxisomal proliferator-activated receptor γ (PPARγ) are activated by 15dPGJ_2_ by this post-translational modification *via* Michael addition [51, 55].

## Mechanisms of action of prostaglandin E_2_

In recent years, several groups have been interested in the role of prostanoids in the regulation of the inflammatory process. Our group focused on studying the effect of PGE_2_ accumulation at sites of inflammation, using cells and animal models deficient in COX-2 or expressing a transgene encoding COX-2, or by administering selective COX-2 inhibitors (called generically coxibs [56–58]), but maintaining the activity of COX-1, an enzyme that contributes to the synthesis of prostanoids in healthy conditions [40, 45, 50, 59, 60].

**Fig. 3:**
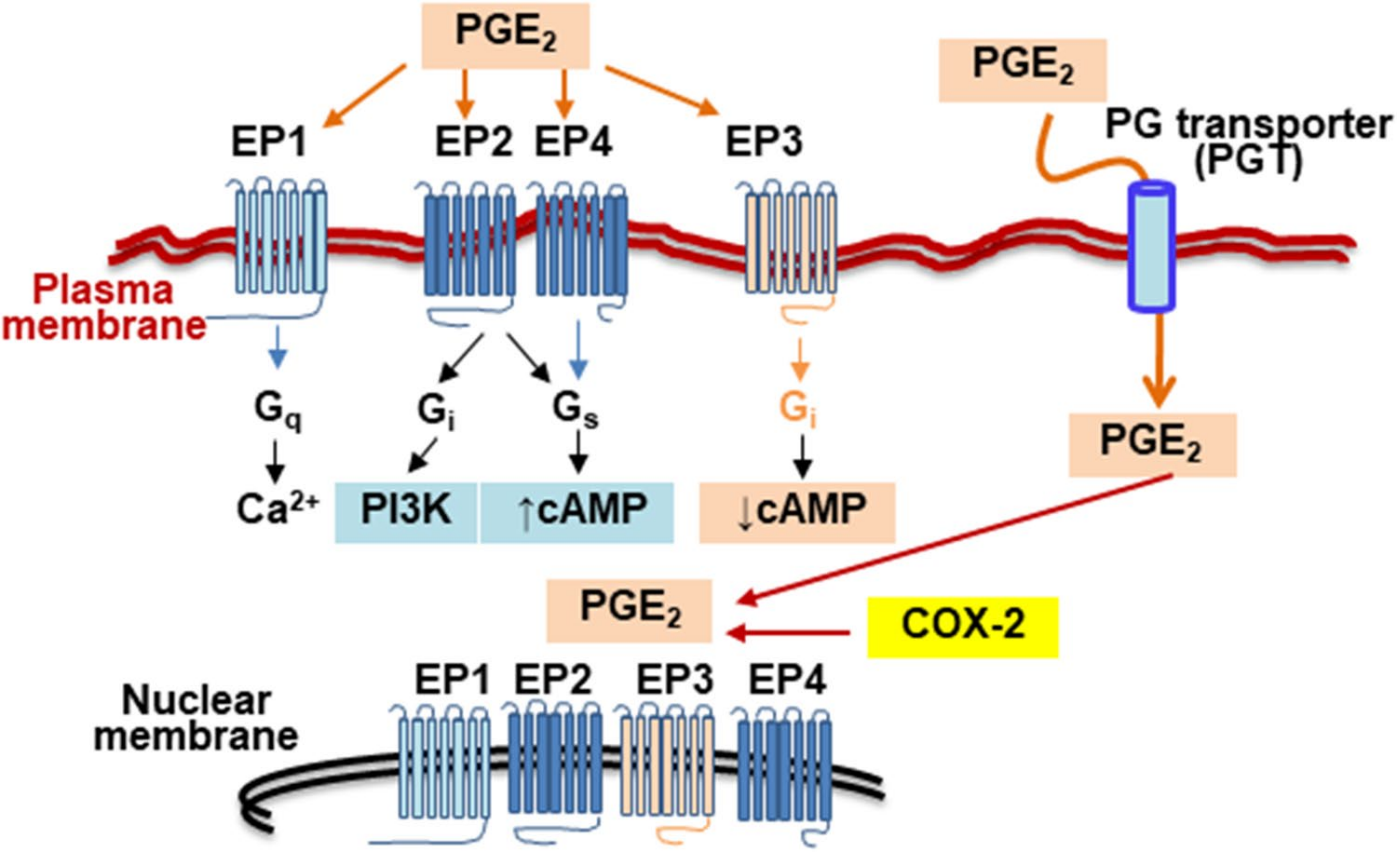
Signaling in response to PGE_2_ biosynthesis. High throughput biosynthesis of PGE_2_ is produced after the expression of COX-2 and activity of the prostaglandin E synthase. PGE_2_ can be exported by the cells and act as an agonist of EP1 to EP4 receptors. Each EP receptor is coupled to specific G proteins that mediate their action. In addition to these plasma membrane receptors, PGE_2_ can be incorporated into the cell via the PG transporter (PGT). In PGE_2_ synthesizing cells, the intracellular presence of this PG can act on EP receptors present at the nuclear membrane. However, the role of these nuclear EP receptors and the mechanisms of signaling are poorly characterized

Regarding the mechanism of action, PGE_2_ binds to and activates specific G protein-coupled membrane receptors called E-type PGE_2_ receptors (EP receptors; Fig. 3). Four different receptors, EP1 to EP4, have been identified from a biochemical and pharmacological point of view [44, 61]. Interestingly, these receptors are not exclusively expressed on the plasma membrane, but also on other intracellular membranes, such as the nuclear membrane [62]. Activation of EP1 promotes the mobilization of intracellular Ca^2+^ stores through activation of the phosphoinositide 3-kinase pathway. This transient change in cytoplasmic Ca^2+^ has an impact on ionic fluxes, cellular metabolism and organelle function (i.e., mitochondria), and activates Ca^2+^-dependent enzymes, such as various isoforms of protein kinase C (PKC). Therefore, PGE_2_ induces Ca^2+^- and PKC-dependent effects in cells expressing EP1 [63, 64]. A relevant fact of EP1 is that the expression profile in cells is different between humans and rodents, which makes it difficult to translate the results between different species [65].

The binding of PGE_2_ to EP2 and EP4 receptors promotes the dissociation of the Gαs/Gβγ complex from the G protein-coupled receptor. The Gαs subunit stimulates adenylate cyclase activity, which increases the intracellular levels of cyclic AMP (cAMP) and, therefore, activates the protein kinase A-dependent pathway [61]. However, EP2 and EP4 have partially non-overlapping functions: EP2 is mainly involved in smooth muscle cell relaxation, whereas EP4 activation exhibits pro- and anti-inflammatory functions ranging from vasodilation to angiogenesis, and metastasis progression [66, 67]. Unlike EP2/EP4, activation of EP3 leads to a reduction in intracellular cAMP levels [68]. These EP receptors are expressed on various cell types and provide the basis for therapeutic interventions, using selective agonists and antagonists. However, in addition to EP-mediated effects, PGE_2_ can exert other actions, either by accessing the cytoplasm or through binding to additional receptors, for example through purinergic signaling, although these mechanisms are less characterized, which explains the effects independent of pharmacological targeting of the EP receptors [69].

## Purinergic signaling in inflammation

Inflammation involves a large number of molecules, including cytokines, chemokines, prostanoids, and extracellular nucleotides that are released during inflammation and activate myeloid and lymphoid immune cells [5, 48, 70–73]. Extracellular nucleotides (i.e. ATP and UTP) have been recognized as a new class of innate immune regulators that act through the P2 receptors and modulate the inflammatory reaction [74–78]. These extracellular nucleotides, which are released at sites of inflammation due to infection or cell damage, contribute to immune cell activation, including cytoskeleton reorganization, cell migration, phagocytosis and exocytosis [72]. Extracellular nucleotides also exert tissue-specific actions. For example, in the brain, they have been associated with different pathologies affecting immune cells (microglia), such as neuropathic pain; indeed, targeting extracellular nucleotide signaling is a pharmacological therapeutic tool that is being investigated in clinical trials [76, 79–83]. Purine and pyrimidine nucleotide receptors are involved in many neuronal and non-neuronal mechanisms: in short-term signaling, they are involved in the regulation of neurotransmission, neuromodulation of inflammation and neurosecretion, promotion of platelet aggregation and vasodilation; and in long-term actions, they are associated with cell proliferation, differentiation, motility, cell-death in development and regeneration.

Currently, the accepted P2Y receptors are P2Y_1_, P2Y_2_, P2Y_4_, P2Y_6_, P2Y_11_, P2Y_12_, P2Y_13_ and P2Y_14_ [84]. Among the metabotropic P2Y receptors, P2Y_2_, P2Y_4_ and P2Y_6_ are activated by uridine and adenine nucleotides [72, 74, 75, 78] and are coupled to phospholipase C (PLC) activation. As a consequence of the release of nucleotides into the extracellular medium, the agonistic action on P2Y receptors promotes an increase in the intracellular concentration of diacylglycerol (DAG) and inositol triphosphate (IP3), which induces the release of calcium from intracellular stores and the activation of several signaling pathways [85, 86]. P2Y receptors are expressed on various cell types and are functionally relevant in the activation of resident and circulating immune cells [5, 24, 71, 74, 87–90].

## Crosstalk between PGE_2_ and P2 receptors in macrophages

The interaction between purinergic signaling and prostanoids has been described in different cell types. In macrophages, exposure to UTP increases the expression of COX-2, and nitric oxide synthase 2 (NOS2) under pro-inflammatory conditions [88, 91]. Macrophages can be polarized into pro-inflammatory (‘classically activated’ or M1, using microbial stimuli such as LPS, or cytokines such as IFNγ) or anti-inflammatory/pro-resolving phenotypes (‘alternatively activated’ or M2, using IL4 and/or IL13 as stimuli) [92–103]. Because macrophages can adopt different functional profiles this crosstalk between PGs and P2 signaling can contribute to the polarization of these cells. Therefore, the activation of P2 receptors helps to modulate the function of macrophages in the context of the environmental signals that govern the fate of the inflammatory response.

The presence of locally elevated concentrations of extracellular ATP promotes the activation of the P2X_7_ receptor, while UTP and UDP, and lower concentrations of ATP act mainly through P2Y_2_, P2Y_4_ and P2Y_6_, respectively [77, 78, 85, 104–106]. Nevertheless, the contribution of P2Y_2_/P2Y_4_ or P2Y_4_/P2Y_6_ heterodimers can also be considered in this regulatory hub [107, 108].

The signaling through the P2X_7_ receptor in macrophages is by far the most studied purinergic pathway. This is because P2X_7_ receptor activation participates in the regulation of several stress signal pathways and, more importantly, activates the NLRP3 inflammasome cascade [109–111]. It is well known that P2X_7_ activation by ATP contributes to the regulation of the innate response in macrophages: it favors the host defense against intracellular pathogens, an effect that is triggered by the release of reactive oxygen and/or nitrogen species [112, 113]. In addition to this, the activation of the NLRP3 pathway promotes the maturation of pro-inflammatory cytokines (i.e., IL-1β and IL-18), and an increase in the PGE_2_ levels. The pathways involved include a rise in Ca^2+^ influx and the activation of the MAP kinase signaling pathways [13, 16, 111].

Interestingly, the crosstalk between P2Y receptors and PGE_2_ has also been reported in macrophages from P2X_7_ receptor-deficient mice, or after inhibition of the receptor with Brilliant Blue G as well as with the receptor antagonist A 438079, which indicates that the interaction between P2Y receptors and PGE_2_ is independent of P2X_7_ receptors [24, 71, 88, 114, 115]. Furthermore, macrophages challenged with specific agonists of the P2X_7_ receptors did not show the inhibitory effect of PGE_2_ on Ca^2+^-mobilization [71]. Regarding the role of the polarization phenotype of macrophages on the expression levels of purinergic receptors, M1 and M2 differentiated cells exhibit similar values, both in RNA and protein levels. However, pro-inflammatory macrophages display rapid and time-dependent repression of the levels of the downstream receptor-associated phospholipase C β1 and β2 isoenzymes, which contribute to the reduced signaling dependent on P2Y receptor activation [116, 117].

The effect of extracellular ATP on the progression of the anti-inflammatory phenotype in macrophages does not involve P2Y/P2X receptor-mediated processes but rather depends on pyrophosphate ATP bonds. The pathways involved promote a reorganization of the actin cytoskeleton that favors the clustering of these actin filaments, which ultimately contribute to the clustering and organization of the NLRP3 inflammasome complex. In addition, the participation of ectonucleotidases seems to contribute to the transition of macrophages from a pro-inflammatory (M1) to an anti-inflammatory (M2) phenotype. This transition is believed to facilitate the resolution of the inflammatory reaction accomplished by macrophages [118–120].

Interestingly, unlike naïve and M2 polarized macrophages, M1 cells do not display the inhibitory effect of PGE_2_ on Ca^2+^ mobilization [24, 71]. These polarization specificities were observed in both rodent and human macrophages. As for the mechanism by which M1 macrophages fail to show this PGE_2_-dependent P2Y desensitization, it has been shown to occur at least two hours after the pro-inflammatory challenge. This suggests that this is not the result of the rapid signaling elicited after TLR4 and/or pro-inflammatory cytokine receptors engagement, but rather is due to secondary events in the signaling process. From a mechanistic point of view, the sustained response to P2Y receptors in the presence of PGE_2_, as occurs in M1 macrophages ensures the activity of the purinergic signaling in the early steps of inflammation [71, 88, 91, 115, 121]. As an extension, in platelets, a cross-desensitization between ADP and the thromboxane receptor signaling has been reported [122, 123]. All of these interactions play an important role in several inflammatory and degenerative disorders, such as multiple sclerosis, amyotrophic lateral sclerosis and Alzheimer´s disease [124–126]. Indeed, in these pathologies, extracellular ATP exerts pro-inflammatory actions that cause the release of cytokines and the production of PG. Interestingly, this modulation could play an important role in the anti-inflammatory effects of PGE_2_.

A relevant aspect in this context of the heterogeneity of P2Y/P2X receptors is the possible crosstalk between the P2X and P2Y receptor families [127–129]. An example is the synergism between both families in the activation of dendritic cells, which are necessary for the efficient initiation of immune responses [130]. In addition to antigens, the presence of P2 agonists released by necrotic cells results in a synergistic activation and maturation of dendritic cells, and therefore, in more efficient signaling in T cells, leading to increased expression of pro-inflammatory mediators and adhesion molecules.

## Molecular mechanisms involved in PGE_2_-P2Y receptor crosstalk

**Fig. 4:**
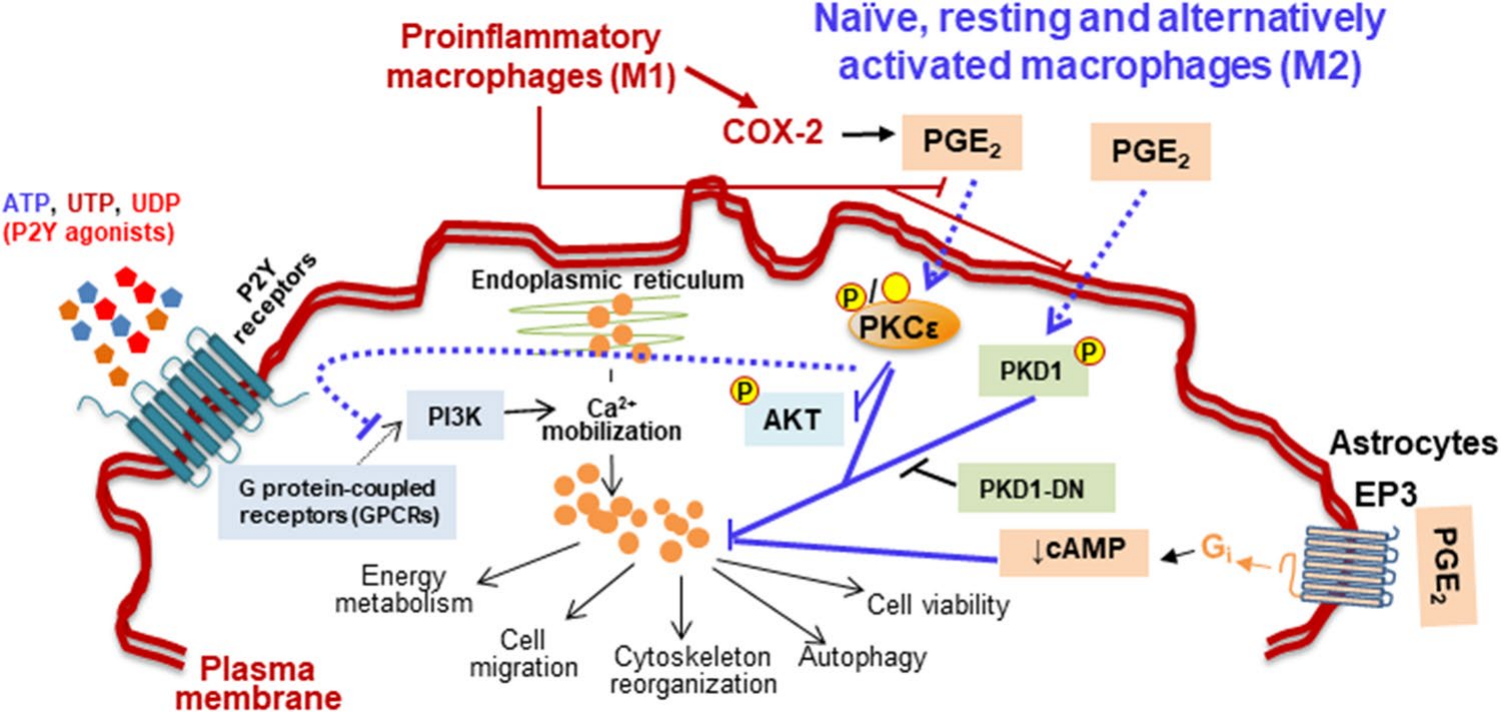
Crosstalk between PGE_2_ and P2Y receptors in macrophages. Pro-inflammatory macrophages express high levels of COX-2 that promote a rapid increase in PGE_2_ synthesis and release. In pro-inflammatory macrophages (M1-type), PGE_2_ is unable to affect the signaling of P2Y receptors. However, naïve, resting, or alternatively activated macrophages (M2-type) exhibit an impaired P2Y receptor signaling that results in a blockade of Ca^2+^-dependent mobilization. This inhibitory effect of PGE_2_ depends on the activities of PKD1 and PKCε and interferes with the different pathways modulated by the transient increase in Ca^2+^ due to P2Y agonists. In cerebellar astrocytes the EP3 receptor is also involved. *Red lines and arrows*, pro-inflammatory pathways; *blue lines and arrows*, resting and anti-inflammatory pathways. PKC, protein kinase C; PKD, protein kinase D; PKD-DN, a dominant-negative form of PKD; AKT, protein kinase B; P, phosphorylation

The pathways involved in the crosstalk between P2Y receptors and PGE_2_ on macrophages have been established using biochemical (inhibitors and activators of signal transduction pathways), pharmacological (mainly through the use of agonists and antagonists of the EP and P2Y receptors) and genetic (cells lacking P2X_7_ receptor or COX-2; expressing a COX-2 transgene or expressing different constructs of the proteins that participate in the signal-transduction pathways) approaches [24, 71, 88, 115, 131, 132]. Based on the data from these different strategies it was concluded that PKD1 phosphorylation at S916 is a necessary condition to suppress PGE_2_-dependent UTP-mediated Ca^2+^-mobilization. In contrast, selective inhibition of PKD1 is sufficient to attenuate the effect of PGE_2_ on P2Y signaling. PKDs are ubiquitously expressed and regulate various cellular processes, including oxidative stress, gene expression, cell survival, vesicle trafficking and, interestingly, P2X_7_ signaling, although their precise function in macrophages remains poorly characterized. Analysis devoted to identifying the PKD isoform(s) involved in this P2Y crosstalk showed that PKD1, which is regulated by extracellular ligands in macrophages, is specifically targeted [24, 71]. Furthermore, overexpression of PKD1 reduced the effect of UTP on Ca^2+^ mobilization but when a vector encoding a catalytically inactive kinase of PKD1 was expressed, the response to UTP persisted and the inhibitory effect of PGE_2_ was abolished (Fig. 4) [71].

In fact, an association of PKD1 with TLR9 and, in general, with the MyD88-dependent pro-inflammatory innate immune responses has been described [133, 134]. Additionally, PKCδ activation has been reported to act as an upstream PKD1 activation step. However, transfection of macrophages with constitutively active PKCδ constructs did not mimic the effects of PGE_2_ on UTP-dependent Ca^2+^ mobilization. However, expression in macrophages of a constitutively active PKCε, but not of other classical, new, or atypical PKCs, was sufficient to mimic the effects of PGE_2_ on P2Y receptors in terms of Ca^2+^ mobilization [71].

Regarding the role of macrophage polarization in this PGE_2_-P2Y crosstalk, naïve and anti-inflammatory/pro-resolving (M2) macrophages show this inhibitory interaction, but it was not observed in those that were polarized to M1 pro-inflammatory cells. Under these M1 conditions, PGE_2_-dependent phosphorylation of PKD1 at S916 is not observed, while naïve and M2 macrophages exhibit this PKD1 phosphorylation [24, 71]. This phosphorylation of PKD1 at S916 has been reported to correspond to a fully activated PKD1. Moreover, activation of PKD1 has been associated with the response to upstream PKCs and/or activation of G-proteins and various receptor-associated tyrosine kinases [135]. The PGE_2_-dependent activation of PKD1 promotes DAGs release not only at the plasma membrane level but also from other compartments, such as the endoplasmic reticulum and the Golgi apparatus. Interestingly, PKD activation plays a role in the crosstalk between P2Y and P2X receptors (Fig. 4). In line with this, P2X_4_ receptor signaling favors the activation of phospholipase A2 (PLA2) and, in turn, the supply of substrates for COX-2 and, therefore, the increase in the release of PGE_2_ that participates in the intercellular crosstalk between P2X and P2Y receptors [107, 136].

The regulation of P2Y activity in macrophages, which involves the participation of PGE_2_, has functional implications in the basic biological responses of these cells, such as metabolic activation and migration. In this regard, cell migration contributes to normal development and differentiation. Recent data indicate that extracellular nucleotides can regulate the migration and attachment activities of “professional phagocytes” (macrophages, neutrophils and microglia) and other cell types (i.e., fibroblasts, endothelial cells, neurons and keratinocytes) [137–139]. From a functional point of view, it has been shown that PGE_2_ inhibits P2Y-dependent macrophage migration, even in the presence of other chemoattractants. These chemotactic actions are common for several P2Y receptors, such as P2Y_2_, P2Y_4_, and P2Y_6_ [140–142]. These observations are consistent with the fact that P2 receptors participate in a wide range of phagocytic and chemotactic actions, as described for P2Y_2,4,6_ receptors in the phagocytosis of apoptotic bodies by microglial cells. In addition to these signaling mechanisms, the EP3 receptors have been involved in the impairment of Ca^2+^-mobilization by PGE_2_ in cerebellar astrocytes [88].

Interestingly, PGE_2_ promotes the internalization of P2Y_4_ in fibroblasts transfected with COX-2, an effect that is suppressed after the inhibition of COX-2 with the coxib DFU [24]. Moreover, the blockade in Ca^2+^-mobilization by PGE_2_ has an important consequence in terms of the activation of different signaling pathways in fibroblasts, including activation of various PKCs and the energetic metabolism via activation of AMP-dependent protein kinase (AMPK) and inhibition of acetyl-CoA carboxylase (ACC) [24, 71]. Again, this regulatory network is suppressed when fibroblasts are in an inflammatory environment. Recent trends in tissue repair of inflammatory lesions have focused on the interaction between stromal cells, such as macrophages and fibroblasts. Based on these observations, it can be proposed that targeting the stromal microenvironment is likely to be an important and promising strategy for future anti-inflammatory and pro-resolution therapies.

In summary, the translation of basic studies on the interactions between prostaglandin synthesis and the signaling through P2Y and P2X receptors in the immune system to clinical trials can result in the development of new therapeutic options to modulate the course of inflammatory diseases.

## Data Availability

Data and comments that support this study are available from the corresponding authors upon request.

## Abbreviations

cAMP: Cyclic AMP
DAMPs: Damage-Associated Molecular Patterns
15dPGJ_2_: 15-Deoxy-Δ.12,14-Prostaglandin J_2_
DAG: Diacylglycerol
DFU: 5,5-Dimethyl-3-(3-fluorophenyl)-4-(4-methylsulphonyl)phenyl-2(5H)-furanone
IFNγ: Interferon-γ
IL: Interleukin
LPS: Lipopolysaccharide
MyD88: Myeloid Differentiation Primary Response 88
NLRP3: NLR Family Pyrin Domain Containing 3
NF-κB: Nuclear Factor κB
PAMPs: Pathogen-Associated Molecular Patterns
PL: Phospholipase
PG: Prostaglandin
PGE_2_: Prostaglandin E_2_
EP: Prostaglandin E_2_ Receptor
PKC: Protein kinase C
PKD: Protein kinase D
P2: Purinergic Receptors
TLR: Toll-Like Receptor
